# Evaluation of CD10 Expression in Endometrial Carcinoma: A Systematic Review

**DOI:** 10.22034/ijp.2026.2074360.3564

**Published:** 2026-08-01

**Authors:** Sara Ashtari, Amir Ebrahimzadeh Pasha, Tima Bashar Awad, Masoud Rezaei, Mohammad Mahdi Mehrabi

**Affiliations:** 1 School of Medicine, Tehran University of Medical Sciences, Tehran, Iran; 2 School of Medicine, Kerman University of Medical Sciences, Kerman, Iran; 3 Research Center for Hydatid Disease in Iran, Kerman University of Medical Sciences, Kerman, Iran; 4 Faculty of Medicine, Shahed University, Tehran, Iran; 5 Urology Research Center, Tehran University of Medical Sciences, Tehran, Iran

**Keywords:** CD10, Endometrial Carcinoma, Immunohistochemistry, Biomarker, Mesonephric-Like Adenocarcinoma, Endometrium

## Abstract

**Background & Objective:**

CD10 is a membrane-bound metalloproteinase that plays a role in tumor progression and differentiation. Its diagnostic significance in endometrial carcinoma, especially in distinguishing between histological subtypes and invasion patterns, is still being studied. This systematic review aims to compile evidence on CD10 expression in endometrial carcinoma, focusing on its prevalence, staining patterns, and correlations with clinicopathological features.

**Methods:**

A comprehensive search was conducted across PubMed, Scopus, and Embase in March 2025, yielding 17 eligible studies. Data on CD10 expression, histological subtypes, staining localization, and associations with tumor grade, stage, and diagnostic utility were qualitatively synthesized. The Newcastle-Ottawa Scale was used to assess the quality of the studies.

**Results:**

CD10 expression was predominantly observed in the stromal compartment of endometrioid carcinomas, with a periglandular or “fringe-like” pattern. Stromal expression levels were consistently high, whereas epithelial expression varied significantly. An inverse correlation between CD10 expression and tumor grade was observed, with lower expression in higher-grade tumors. The role of CD10 in differentiating myoinvasion from adenomyosis was limited, as both conditions exhibited overlapping staining patterns. Luminal epithelial staining in mesonephric-like adenocarcinomas was not exclusive, further complicating its diagnostic value.

**Conclusion:**

CD10 is a useful marker of stromal tissue in endometrial carcinoma and may aid in distinguishing histological patterns and mimics. However, its diagnostic and prognostic value is constrained by nonstandardized methods and overlapping expression profiles. Standardizing IHC protocols and conducting large-scale studies are essential to enhance CD10’s clinical utility.

## Introduction

Endometrial carcinoma (EC) is the most prevalent malignancy of the female reproductive system in developed countries. It ranks as the third most common cancer among women, with a rising incidence(1). Different tumor subtypes exhibit various microscopic and morphological characteristics that are important for tumor staging. According to the FIGO staging system, ECs are classified into nonaggressive histological types (low-grade endometrioid carcinomas, grades 1–2) and aggressive histological types (high-grade endometrioid carcinoma [grade 3], serous, clear cell, carcinosarcoma, mesonephric-like adenocarcinoma, and others)(2). Uterine mesonephric-like adenocarcinoma (MLA) is a rare and aggressive subtype of adenocarcinoma, constituting less than 1% of all malignant tumors in females(3). A precise histopathological diagnosis is important for assessing tumor invasion, grade, and subtype, as it greatly impacts prognosis and treatment decisions(4). However, distinguishing between tumors and benign conditions or recognizing rare tumor subtypes such as MLA can pose significant diagnostic challenges due to their similar morphological features.

Cluster of differentiation 10 (CD10), known as CALLA, neprilysin, and neutral endopeptidase, is a type II integral protein found on the cell surface(5,6). This glycoprotein spans the membrane and is responsible for cleaving and inactivating specific peptides, such as bombesin and enkephalins(7). Previous studies have reported that CD10 expression is associated with tumor aggressiveness, differentiation, and biological tumor transformation in several cancers, including melanoma, colorectal cancer, breast cancer, and bladder cancer(8-10). In the endometrium, research indicates that CD10 is expressed in both normal and ectopic endometrium(11,12). Additionally, CD10 is identified as a diagnostic immunohistochemistry (IHC) biomarker for detecting endometrial neoplasms(13).

Numerous studies have explored the expression of CD10 in various types of endometrial neoplasms(14-17). However, the existing literature on CD10 expression in ECs is fragmented, with inconsistencies in study design, sample sizes, histological focus, and interpretation criteria for IHC. To better understand the diagnostic and potential prognostic value of CD10, a systematic evaluation across multiple studies is necessary.

The primary aim of this systematic review was to synthesize the available evidence on CD10 expression in EC, with particular emphasis on its prevalence, cellular localization, and staining patterns across different histological subtypes. The secondary objectives were to evaluate the associations between CD10 expression and clinicopathological features, including tumor grade, FIGO stage, and myometrial invasion; to assess the diagnostic utility of CD10 in distinguishing EC subtypes and benign mimics; and to identify methodological limitations related to IHC protocols, scoring systems, and positivity thresholds that may affect interpretation of the existing evidence.

## Materials and Methods

### Study Design

**Fig. 1:**
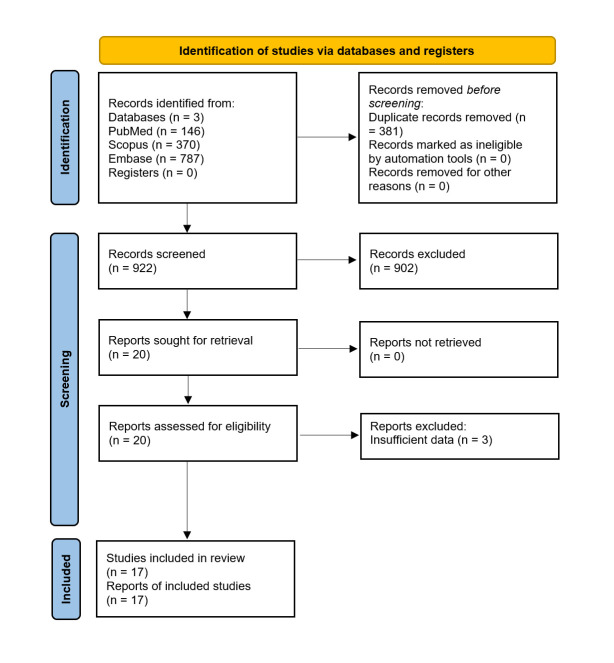
Prisma flow diagram. The flowchart illustrates the selection of studies process.

The study protocol was followed and registered according to the guidelines set forth by the Preferred Reporting Items for Systematic Reviews and Meta-Analyses (PRISMA) 2020 and registered in PROSPERO under the code CRD420251016446(18). Fig 1 illustrates the study selection process. The completed PRISMA 2020 checklist is provided as Supplementary Table 1. Additional details regarding the review process are provided in the following subsection.

### Literature Search Strategy

A comprehensive literature search was conducted in PubMed, Scopus, and Embase in March 2025. The final search was performed on March 25, 2025. No restrictions were applied regarding publication year or date of publication. The search strategy combined controlled vocabulary terms, where available, and free-text terms related to CD10 and endometrial neoplasia. The reference lists of all eligible articles were also manually screened to identify additional relevant studies. Complete database-specific search strings are provided in Supplementary Table 2.

### Eligibility Criteria

Studies were eligible for inclusion if they met the following criteria:

Investigated CD10 expression in EC tissue.

Reported data on expression prevalence, staining patterns, or clinicopathological correlations.

The articles were peer-reviewed original research studies, including cross-sectional, retrospective, and cohort designs.

Were published in English.

### Exclusion Criteria:

Review articles, editorials, case reports, conference abstracts, or animal studies.

Studies that did not perform or report CD10 analysis in EC.

Research focused exclusively on non-endometrial malignancies or cell lines.

Incomplete data or lack of access to the full-text article.

The English-language restriction was applied to ensure accurate assessment of detailed histopathological terminology, immunohistochemical scoring systems, staining localization, and clinicopathological correlations. Because these data require precise interpretation, only studies with reliable full-text translation were included.

### Study Selection

A total of 1303 records were identified through database searches, including PubMed (n = 146), Scopus (n = 370), and Embase (n = 787). After removing 381 duplicate records, 922 records remained. Two reviewers (T.B.A. and A.E.P.) independently screened the titles and abstracts for relevance. Of these, 902 records were excluded based on their relevance, and 20 full-text articles were sought for retrieval. All 20 full texts were successfully retrieved and assessed for eligibility. Among these, 3 studies were excluded at the full-text stage because they did not provide extractable CD10 expression data specific to EC or did not meet the predefined eligibility criteria. As a result, 17 studies met the inclusion criteria and were included in the final systematic review. No additional eligible studies were found after 2024. Disagreements were resolved either by consensus or through consultation with a third reviewer (M.M.M.).

Potential overlap among study cohorts was assessed during full-text review and data extraction by comparing author groups, institutions, countries, recruitment periods, tumor subtypes, sample sizes, and reported clinicopathological characteristics. When studies appeared to include potentially overlapping cases, the study with the most complete and directly relevant CD10 expression data was prioritized for interpretation. Overlapping cohorts were not double-counted when summarizing prevalence, staining patterns, or clinicopathological associations. The included studies did not identify a confirmed duplicate cohort; however, because some older studies did not provide detailed recruitment periods, partial overlap remained possible.

### Data Extraction

Data were extracted using a standardized data extraction form tailored to the study objectives and informed by the PICOS framework. Two reviewers (A.E.P. and S.A.) independently extracted the following information from each eligible study:

First author, year of publication, and country of origin

Study design

Number and types of EC cases, including total sample size and distribution of histological subtypes

Patient demographic information, including age as mean, median, or range when reported.

CD10 expression data, including the number and percentage of positive cases, the cellular compartment of staining, and detection methods

Staining pattern characteristics, such as diffuse, focal, periglandular, or luminal localization

Criteria used to define CD10 positivity, including thresholds based on intensity, percentage of positive cells, or composite scoring systems, such as H-score and qualitative descriptions

Associations with clinicopathological parameters, such as FIGO stage, tumor grade, presence of myoinvasion, and comparison with other immunomarkers

Statistical results, such as reported P values for correlations between CD10 expression and clinicopathological features, or sensitivity/ specificity for diagnostic discrimination

Author-reported main findings and limitations, and any additional relevant observations or interpretations, such as diagnostic implications and alternative markers suggested

When provided, statistical values, such as P-values for correlations, were noted.

### Quality Assessment

The methodological quality of the included studies was evaluated using a modified version of the Newcastle-Ottawa Scale (NOS). This tool assesses studies across 3 primary domains: Selection (maximum score: 5), Comparability (maximum score: 2), and Outcome (maximum score: 3). Each study was independently reviewed and scored based on the available information. The highest possible score for each study was 10, with higher scores indicating a lower risk of bias and greater methodological rigor(19). Two reviewers (T.B.A. and M.R.) independently evaluated the risk of bias in the studies. In cases of disagreement, a third reviewer (S.A.) checked the data and made the final decision. The modified NOS criteria used in this review are described in detail in Supplementary Table 3.

**Fig. 2:**
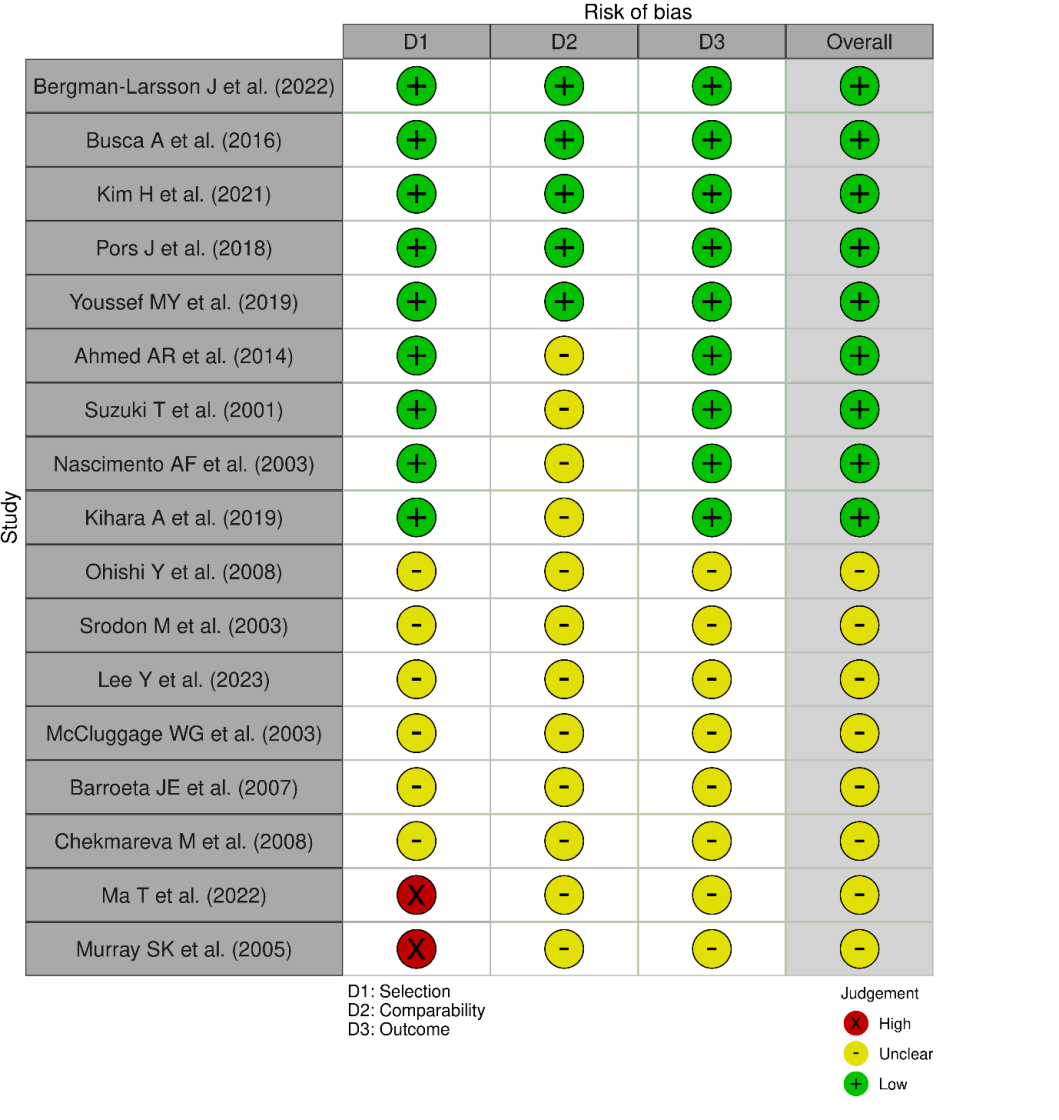
Quality assessment of studies with the NOS scale. D1: selection domain, D2: comparability domain, D3: outcome domain.

The predominance of low-risk studies suggests that the core evidence base is relatively strong, particularly for more recent publications. However, the moderate risks in comparability and outcome domains across many studies introduce potential heterogeneity and limit confidence in results (Fig. 2, Table 1). Future research should prioritize transparent reporting in comparability to enhance methodological quality. This assessment underscores the need for cautious interpretation of the review's results, with higher weight given to low-risk studies in synthesis.

**Table 1:** Quality assessment of studies by the NOS tool

Study (year)	Selection	Comparability	Outcome	Total Score
Bergman-Larsson J et al. (2022)	4	2	3	9
Busca A et al. (2016)	4	2	3	9
Kim H et al. (2021)	4	2	3	9
Pors J et al. (2018)	4	2	3	9
Youssef MY et al. (2019)	4	2	3	9
Ahmed AR et al. (2014)	4	1	3	8
Suzuki T et al. (2001)	4	1	3	8
Nascimento AF et al. (2003)	4	1	3	8
Kihara A et al. (2019)	4	1	3	8
Ohishi Y et al. (2008)	3	1	2	6
Srodon M et al. (2003)	3	1	2	6
Lee Y et al. (2024)	3	1	2	6
McCluggage WG et al. (2003)	3	1	2	6
Barroeta JE et al. (2007)	3	1	2	6
Chekmareva M et al. (2008)	3	1	2	6
Ma T et al. (2022)	2	1	2	5
Murray SK et al. (2005)	2	1	2	5

### Data Synthesis

Given the variability in staining protocols, scoring systems, and reported outcomes, meta-analysis was not feasible. Therefore, data were synthesized qualitatively. Results were organized into 3 thematic categories:

Prevalence and distribution of CD10 expression.

Staining patterns and diagnostic implications.

Associations with clinicopathological features and potential prognostic value.

## Results

### Study Selection and Characteristics

This systematic review included 17 observational studies investigating CD10 expression in EC, published between 2001 and 2024(9,14–17,20–26,28–32). The studies originated from various countries, including Egypt, the USA, Sweden, Canada, South Korea, China, Japan, and the UK. The total number of patient samples analyzed across these studies varied considerably, ranging from 3 to 262 relevant cases per study, focusing on specific endometrial pathologies, although one study included a larger cohort of 463 diverse gynecological adenocarcinomas. Participant age information was reported variably, often as means or medians, typically falling within the 50s and 60s. Notably, no studies meeting the inclusion criteria specifically focused on the prevalence or significance of CD10 expression in endometrial hyperplasia were identified in the extracted data; the focus was predominantly on various types of EC. Detailed characteristics of the included studies are presented in Table 2.

**Table 2:** Characteristics of Included Studies

First Author	Year	Country	Main Histological Type(s) Studied	Total Samples (n)	Age Summary (year)
Ahmed AR	2014	Egypt	Endometrioid (n=23), Serous (n=5).	28	Mean 52.5 ± 11.2
Barroeta JE	2007	USA	Endometrioid	13	NR
Bergman-Larsson J	2022	Sweden	Endometrioid Carcinoma, Metastases	262	NR
Busca A	2016	Canada	Endometrioid (Invasive vs Adenomyosis)	25	Mean 62.5
Murray SK	2005	Canada	Endometrioid (with sex cord-like features)	10*	Median 52
Kim H	2021	South Korea	MLA	21	Median 59
Ma T	2022	China	MLA	3	Mean 68
Kihara A	2019	Japan	Endometrioid (Myoinvasive vs APA)	30	Mean 37 Median 35
Lee Y	2024	South Korea	Low-grade Endometrioid Carcinoma (EEC)	50	Median 53
McCluggage WG	2003	UK	Endometrioid Adenocarcinoma (n=9), Mesonephric Adenocarcinoma (n=3)	12	NR
Youssef MY	2019	Egypt	Endometrioid (n=32), Villoglandular (n=4)	36	Mean 60.97 ± 8.18
Suzuki T	2001	Japan	Endometrioid	32	Mean 59.3
Srodon M	2003	USA	Endometrioid (Invasive vs Adenomyosis involvement)	27	NR
Pors J	2018	Canada	Various Endometrial/Cervical Adenocarcinomas	77 (Endometrial)	Mean 61
Ohishi Y	2008	Japan	Myoinvasive Endometrioid Carcinoma	19	Mean 60 Median 55
Nascimento AF	2003	USA	Endometrioid (Invasive vs Adenomyosis involvement)	39	Median ~60
Chekmareva M	2008	NR	Mucinous (n=30), Microglandular (n=10) Adenocarcinoma	40	Mean ~67-70

NR: Not Reported. Sample counts reflect the primary endometrial carcinoma cohorts analyzed for CD10 as per the data extraction sheet. *Only 10 had CD10 data

### Antibody Clones, Dilutions, and Detection Methods

The majority of studies utilized mouse monoclonal antibodies for CD10 detection, particularly the 56C6 clone. Other clones, such as SP67 and M7308, were used in a limited number of studies. The dilution protocols varied widely, ranging from 1:5 to 1:100. All studies employed IHC as the detection method (Table 3).

**Table 3:** CD10 Antibody, Cutoff, and Scoring Criteria Across Included Studies

Author (Year)	Antibody Clone / Vendor	Dilution	Scoring Method	Cutoff / Criteria	Compartment Assessed
Ahmed (2014)	Monoclonal /56C6, Thermo	NR	Intensity (0–3)	Weighted score >0	Epithelial, Stromal
Barroeta (2007)	Monoclonal /56C6	1:50	Percentage	>5% cells	Stromal
Bergman-Larsson (2022)	Monoclonal anti-CD10	NR	Qualitative (Weak/Strong)	>0% cells	Stromal
Busca (2016)	NR	NR	Composite (Intensity + % Pos)	Total score >3	Stromal
Murray (2005)	NR	NR	Any positive staining	Presence/absence	Epithelial
Kim (2021)	Monoclonal anti-CD10 /56C6	1:100	Percentage (H-score)	>5% cells	Epithelial
Ma (2022)	Monoclonal anti-CD10/SP67	NR	Pattern (luminal)	Any positive luminal	Epithelial
Kihara (2019)	Monoclonal anti-CD10/56C6	1:100	Intensity (0–3+)	Pattern-based	Stromal
Lee (2024)	Monoclonal anti-CD10/56C6	1:100	Composite (0–2+, %)	Any positive staining	Epithelial
McCluggage (2003)	Monoclonal /56C6	1:5	Percentage, Pattern	>10% cells	Epithelial, Stromal
Youssef (2019)	monoclonal mouse anti-CD10/ M7308	NR	Intensity (1 or 2)	Intensity 1/2 = positive	Stromal
Suzuki (2001)	monoclonal mouse anti-CD10/56C6	1:50	Intensity (>1)	Score >1	Stromal
Srodon (2003)	monoclonal mouse anti-CD10	NR	Any positive perinest staining	Any positive perinest	Stromal
Pors (2018)	NCL-L-CD10-270	1:50	Percentage	>1% positive cells	Epithelial, Stromal
Ohishi (2008)	monoclonal anti-CD10/56C6	1:100	Pattern only	“Fringe-like” pattern	Stromal
Nascimento (2003)	mouse monoclonal antibody/56C6	1:80	Presence/absence	Any positive around glands	Stromal
Chekmareva (2008)	monoclonal anti-CD10	NR	Intensity (0–3)	>0 score	Stromal

### Scoring Methods and Methodological Heterogeneity

CD10 expression was assessed using 3 primary scoring methods: intensity-based scoring, percentage-based scoring, and composite scoring. Intensity-based scoring categorized staining as negative, weak, moderate, or strong(9,28,29). In percentage-based scoring, positivity was defined based on the percentage of stained cells, with thresholds varying between 1% and 10%(14,22,25). In composite scoring, some studies combined both intensity and percentage positivity in their assessments(15,20,23).

A summary of the methodologies used in these studies is presented in Table 3.

### Prevalence and Patterns of CD10 Expression

The prevalence of CD10 expression varies significantly based on the histological subtype, the cellular compartment being assessed (stromal vs epithelial), and the criteria for positivity applied. Stromal CD10 expression is consistently high across studies, particularly in endometrioid carcinomas, with positivity rates often exceeding 85%. For example, Srodon et al (2003) reported 100% stromal positivity, Suzuki et al (2001) found 94% positivity, and Youssef et al (2019) documented 92% positivity. The predominant stromal staining patterns included diffuse, patchy, periglandular, and fringe-like distributions(27-30).

In contrast, epithelial CD10 expression displayed considerable variability, ranging from 8% to 93% positivity, with higher rates typically observed in endometrioid carcinoma. Specifically, Ahmed et al (2014) reported 93% epithelial positivity, while McCluggage et al (2003) found 78% positivity. Luminal epithelial staining, which is often associated with MLA, was observed in 81% of cases by Kim et al (2021) and in 100% of cases by Ma et al (2022)(9,22,24,25).

CD10 expression in other histological subtypes exhibited greater variability. Pors et al (2018) reported positivity rates of 14% in grade 1-2 endometrioid carcinomas, 12% in grade 3 endometrioid carcinomas, and 7% in both serous and clear cell carcinomas. Additionally, 35% positivity was observed in carcinosarcomas and 20% in mixed carcinomas. Furthermore, Ahmed et al (2014) found significantly lower CD10 expression in serous carcinoma compared with endometrioid carcinoma. Strong stromal expression was also commonly reported in mucinous and microglandular subtypes(9,31).

The predominant staining pattern in endometrioid carcinoma was stromal, characterized by descriptions of diffuse, patchy, periglandular, or fringe-like staining around invasive glands. When epithelial staining was present, it was primarily membranous or membranocytoplasmic. Although luminal staining is characteristic, it is not exclusive to mesonephric-like differentiation, as noted in the studies by Kim et al (2021), Ma et al (2022), and Pors et al (2018)(9,14-17,20-22,24,25). Details on prevalence and patterns are summarized in Table 4.

**Table 4:** CD10 Prevalence and Staining Patterns in Endometrial Adenocarcinoma Subtypes

First Author	Histological Type(s)	CD10+ / Total (%)	Staining Pattern(s) Reported	Positivity Criteria Summary
Ahmed AR	Endometrioid, Serous	26 / 28 (93%)	Membranous or Membrano-cytoplasmic (Epithelial)	Weighted score > 0
Barroeta JE	Endometrioid (stroma)	11 / 13 (85%)	Cytoplasmic (Stromal)	>5% Stromal Cells
Bergman-Larsson J	Endometrioid	225 / 262 (86%)	Stromal (surrounding glands/tumor)	Qualitative (Weak/Strong >25%)
Busca A	Endometrioid	25 / 25 (100%)	Stromal (Patchy/Diffuse/Circumferential)	Total Score > 3 (Intensity + Distribution)
Murray SK	Endometrioid (sex cord-like)	1 / 10 (10%)	Glandular component (Epithelial)	Any positive staining
Kim H	Mesonephric-like	17 / 21 (81%)	Primarily Luminal; some Basolateral/Circumferential (Epithelial)	>5% Positive Tumor Cells (H-Score)
Ma T	Mesonephric-like	3 / 3 (100%)	Focal Luminal (Epithelial)	Any positive staining
Kihara A	Endometrioid (Myoinvasive)	22 / 30 (73%)	Stromal (Periglandular 'fringe')	Score >= 1+ (% Positive Cells)
Lee Y	Low-grade Endometrioid	4 / 50 (8%)	Luminal, Membranous/Cytoplasmic (Epithelial)	Any positive staining
McCluggage WG	Endometrioid, Mesonephric	7/9 EEC (78%) 2/3 Mesonephric (67%)	Membranous, Luminal, Cytoplasmic, Nuclear (Epithelial/Stromal)	>10% Positive Cells (Implied)
Youssef MY	Endometrioid, Villoglandular	33 / 36 (92%)	Membrano-cytoplasmic, Linear pattern (Stromal)	Intensity Score 1 or 2
Suzuki T	Endometrioid	30 / 32 (94%)	Stromal cells only	Intensity Score > 1
Srodon M	Endometrioid (Invasive/Adenomyosis)	27 / 27 (100%)	Stromal (around carcinoma nests)	Any positive staining around nests
Pors J	Various (Endo G1-2, G3, Serous, CCC, CS, Mixed)	14% (G1-2) 12% (G3) 7% (Serous/CCC), 35% (CS)	Cytoplasmic and Luminal (Epithelial/Stromal)	>1% Positive Cells
Ohishi Y	Myoinvasive Endometrioid	NR positive count (Mean 74% nests surrounded)	Stromal (Periglandular 'fringe')	Not mentioned
Nascimento AF	Endometrioid (Invasive/Adenomyosis)	36 / 39 (92%)	Stromal (Circumferential or Focal around glands)	Any positive staining around glands
Chekmareva M	Mucinous, Microglandular	40 / 40 (100%)	Cytoplasmic (Stromal)	Intensity Score > 0

Percentages calculated where data allowed. Staining patterns indicate the primary location reported. Positivity criteria varied significantly.

### Association With Clinicopathological Features and Diagnostic Utility

Several studies investigated the association between CD10 expression and clinicopathological parameters, primarily in endometrioid carcinoma. A recurrent finding was an inverse association between CD10 expression, often stromal or overall epithelial/stromal, and tumor grade. Ahmed et al (2014) reported significantly lower expression in grade 3 compared with grade 1/2 tumors (P = 0.017/0.016), and Youssef et al (2019) found a significant decrease with increasing grade (P = 0.001). Suzuki et al (2001) also observed an inverse correlation between stromal CD10 score and grade, with a significant decrease in grades 2 and 3 compared with normal endometrium (P < 0.05)(9,28,29).

Findings regarding FIGO stage were inconsistent. Youssef et al. (2019) reported a significant decrease in CD10 expression with advancing stage (P = 0.001), whereas Ahmed et al. (2014) and Suzuki et al. (2001) found no significant association. Ahmed et al. (2014) noted lower expression in serous carcinoma compared with endometrioid carcinoma (P = 0.048), and Pors et al. (2018) observed the highest rates in carcinosarcomas among various subtypes. Youssef et al. (2019) also reported a positive correlation between CD10 and E-cadherin expression (P = 0.001)(9,17,28,29).

The utility of CD10 in distinguishing myoinvasion from carcinoma involving adenomyosis yielded complex results. While Srodon et al. (2003) found CD10 staining around carcinoma nests in both scenarios, rendering it nondiscriminatory, Nascimento et al. (2003) observed that the absence of stromal CD10 staining around glands was strongly suggestive of myoinvasion, seen in 35.8% of invasive cases vs. 0% of adenomyosis involvement. However, the presence of CD10 staining occurred in both invasion and adenomyosis involvement, limiting its discriminatory power when positive. Busca et al. (2016) found lower CD10 stromal scores in invasive carcinoma compared with adenomyosis involvement (P = 0.04) but noted the marker's low specificity and suggested IFITM1 might be superior. Kihara et al. (2019) and Ohishi et al. (2008) proposed that the pattern of stromal staining, a periglandular “fringe” in carcinoma vs. diffuse in associated benign conditions such as APA, might be more informative than expression levels alone, although Kihara et al. found no significant difference in overall expression levels between myoinvasive carcinoma and APA, suggesting stromal p16 might be a better discriminator(16,20,21,26,27).

CD10 was confirmed as a useful marker for identifying endometrial stroma, potentially aiding in differentiating it from endocervical stroma. While CD10 expression, particularly luminal staining, was common in MLA, it was noted not to be entirely specific, as it could be seen in other EC types. Some studies suggested that other markers, such as GATA3 or calretinin, might offer better specificity for mesonephric differentiation(14,15,17,22-25). Limited data addressed prognostic significance. Kim et al. (2021), in a small cohort of MLA, found no significant association between the proportion of CD10 staining and recurrence or disease-free survival (P = 1.0)(22). Key associations are summarized in Table 5.

**Table 5:** Association of CD10 Expression with Clinicopathological Features and Diagnostic Utility

First Author	Feature Investigated	Key Finding / Association (p-value)	Staining Compartment Assessed
Ahmed AR	Grade	Inverse assoc. (p=0.015); Higher G1/G2 vs. G3 (p=0.017/0.016)	Epithelial/Overall
Ahmed AR	Stage	No association	Epithelial/Overall
Ahmed AR	Histological Type	Lower in Serous vs Endometrioid (p=0.048)	Epithelial/Overall
Barroeta JE	Endometrial vs Endocervical	Significant difference (p=0.0004); Useful differentiator	Stromal
Bergman-Larsson J	Endometrial Stroma ID	Combined HOXA11/CD10 signature specific	Stromal
Busca A	Myoinvasion vs Adenomyosis	Lower score in invasion (p=0.04); Absence suggests invasion (High NPV)	Stromal
Kim H	Recurrence/DFS (MLAC)	No significant difference based on CD10 proportion (p=1.0)	Epithelial
Kihara A	Myoinvasion vs APA	Pattern differs ('fringe' vs diffuse); Expression level not significantly different.	Stromal
Lee Y	Mesonephric Markers	No significant association with TTF1, GATA3	Epithelial
McCluggage WG	Mesonephric Specificity	Positive in EEC (78%); Not specific for mesonephric	Epithelial/Stromal
Youssef MY	Grade	Significant decrease with increasing Grade (p=0.001)	Stromal
Youssef MY	Stage	Significant decrease with increasing Stage (p=0.001)	Stromal
Youssef MY	E-cadherin	Positive correlation (p=0.001)	Stromal
Suzuki T	Grade	Inverse correlation; Significant decrease G2/G3 vs Normal (p<0.05)	Stromal
Suzuki T	Stage	No correlation	Stromal
Srodon M	Myoinvasion vs Adenomyosis	Present in both; Not useful to distinguish	Stromal
Pors J	Histological Type	Variable rates (Highest in CS 35%, Lowest in Serous/CCC 7%)	Epithelial/Stromal
Pors J	Mesonephric Specificity	Luminal pattern is rare except in mesonephric; GATA3 is considered better	Epithelial/Stromal
Ohishi Y	Myoinvasion vs APA	'Fringe-like' pattern characteristic of myoinvasion	Stromal
Nascimento AF	Myoinvasion vs Adenomyosis	Absence suggests invasion; Presence is non-discriminatory; Reliably identifies adenomyosis stroma itself.	Stromal

APA: Atypical Polypoid Adenomyoma; CS: Carcinosarcoma; CCC: Clear Cell Carcinoma; DFS: Disease-Free Survival; EEC: Endometrioid Endometrial Carcinoma; MLAC: Mesonephric-like Adenocarcinoma; NPV: Negative Predictive Value.

### Qualitative Meta-Summary

Due to methodological heterogeneity, a quantitative meta-analysis was not feasible. Qualitative synthesis showed the following trends:

Stromal CD10 positivity was consistently high (>90%).

Epithelial CD10 expression showed significant variability (8%–93%).

An inverse association with tumor grade was consistently observed.

Inconsistent correlations with tumor stage and limited diagnostic utility for myoinvasion were noted.

### Publication Bias

Because of substantial heterogeneity in study design, IHC protocols, staining compartments, scoring systems, and reported outcomes, formal assessment of publication bias using funnel plots or regression-based methods was not feasible. Therefore, publication bias was assessed qualitatively.

The available evidence may be affected by selective publication, particularly a tendency to report positive or diagnostically informative findings. Several included studies focused on specific diagnostic contexts in which CD10 was expected to be useful, such as identifying endometrial stroma, distinguishing myoinvasion from adenomyosis or atypical polypoid adenomyoma, and supporting the diagnosis of MLA. This focus may have favored publication of studies showing distinctive staining patterns, significant clinicopathological associations, or potential diagnostic utility, while studies with negative, inconsistent, or nonspecific CD10 findings may be underrepresented.

This concern is reinforced by the small sample sizes in studies of rare histological subtypes, the predominance of retrospective observational designs, and variability in reported outcomes. Some studies emphasized stromal or epithelial positivity, staining pattern, or selected clinicopathological correlations, whereas negative or nonsignificant findings may have been less consistently reported. In addition, restriction to English-language peer-reviewed full-text articles and exclusion of abstracts or unpublished data may have contributed to selective evidence capture.

Overall, publication bias cannot be excluded and may have led to overestimation of the diagnostic or clinicopathological relevance of CD10. Therefore, the findings should be interpreted cautiously and considered hypothesis-generating until confirmed by larger prospective studies using standardized immunohistochemical protocols and complete reporting of both positive and negative findings.

## Discussion

CD10, also known as common acute lymphoblastic leukemia antigen (CALLA), is a cell surface zinc-dependent metalloproteinase(32). CD10 is known for its role in regulating the activity of peptide substrates by reducing their local concentration. Research has indicated that CD10 expression in tumor cells plays a significant role in tumor transformation, progression, and apoptosis. Additionally, intratumoral cells expressing CD10 are associated with tumor progression and aggressiveness in various epithelial malignancies, including gastric carcinoma, colon carcinoma, and breast carcinoma(33-35). In the stromal cells of the endometrium, CD10 is regarded as the most reliable immunomarker. However, not all mesenchymal tumors express this biomarker(36). This systematic review synthesizes the existing literature on CD10 expression in EC, providing a comprehensive understanding of its diagnostic and potential prognostic utility.

CD10 is widely recognized as a sensitive marker of endometrial stroma in both normal and pathological conditions(11,13). In benign conditions, such as proliferative endometrium and endometrial hyperplasia, CD10 consistently exhibits cytoplasmic/membranous staining in stromal fibroblasts(9). However, in premalignant conditions like atypical hyperplasia or endometrial intraepithelial neoplasia (EIN), the stroma maintains high CD10 expression, while decidualized stroma, as seen in the Arias-Stella reaction, often loses CD10 expression(12,37,38). This pattern supports the use of CD10 in differentiating nondecidualized proliferative stroma from its mimics. In atypical polypoid adenomyoma (APA), a benign lesion that can be confused with invasive carcinoma, stromal CD10 staining is either absent or diffuse, in contrast to the periglandular or “fringe-like” pattern observed in invasive endometrioid carcinoma(16,21). This spatial distribution of CD10 may have greater diagnostic relevance than the absolute staining intensity or proportion.

Our review confirmed that stromal CD10 expression is nearly universal in endometrioid carcinoma, with positivity rates consistently exceeding 85%(27,29). The typical staining pattern is periglandular or circumferential, sometimes referred to as “fringe-like,” particularly around infiltrative glandular nests. This morphological feature has been suggested as a histologic hallmark of myoinvasive carcinoma(20). However, despite frequent expression, CD10 alone does not reliably differentiate carcinoma invading the myometrium from carcinoma involving adenomyosis. In contrast to stromal staining, epithelial CD10 expression in EC is highly variable. Some studies report epithelial CD10 positivity rates as high as 93%, while others report rates as low as 8%(9,23). This heterogeneity may reflect differences in histological subtype, tumor differentiation, or criteria for immunohistochemical interpretation. Notably, an inverse relationship between CD10 expression and tumor grade has been consistently observed: higher CD10 levels are more common in nonaggressive tumors (grades 1–2), while aggressive tumors (grade 3) show decreased or absent expression(28,29). This trend supports the hypothesis that CD10 expression reflects the interaction between stromal and epithelial cells, as well as the differentiation status of tumors. As tumors dedifferentiate and adopt a more aggressive phenotype, CD10 expression may decrease. However, the underlying mechanisms for this observation remain unclear and require further molecular investigation.

CD10 expression is significantly lower in aggressive subtypes, such as serous carcinoma, clear cell carcinoma, and carcinosarcoma(17). In contrast, MLA often shows frequent CD10 expression in luminal epithelial compartments, with reports indicating positivity in 81%–100% of cases(22,24). While this luminal staining suggests mesonephric differentiation, it is not wholly specific. CD10 luminal positivity has also been observed in low-grade endometrioid carcinomas(23,25). Therefore, while CD10 alone cannot confirm an MLA diagnosis, it may provide additional diagnostic support when interpreted alongside other markers, such as GATA3, TTF-1, and calretinin(17).

CD10 has been associated with tumor progression in various malignancies, including breast, bladder, and colorectal cancers(8,39,40). In these tumors, high CD10 expression often correlates with invasion, metastasis, and poor differentiation. These parallels raise the possibility that CD10 plays an active mechanistic role in modulating the tumor microenvironment across multiple cancer types(41). However, the biological significance of these findings may vary by tumor type and tissue compartment. In the context of EC, the available evidence does not establish a direct mechanistic role for CD10 in tumor progression. Rather, the included studies suggest that decreased stromal or overall CD10 expression may be associated with higher tumor grade and dedifferentiation in some cohorts(9,28,29,34). These observations should be interpreted cautiously because most included studies were retrospective, used heterogeneous immunohistochemical methods, and did not include molecular or functional validation. Therefore, although CD10 may reflect changes in the tumor–stromal microenvironment, its role in extracellular matrix remodeling, epithelial–mesenchymal transition, or immune regulation remains hypothetical and requires further investigation.

IHC has played a central role in all studies examining CD10 expression. As a widely used technique in pathology, IHC allows for the localization of protein markers within specific tissue compartments, providing both visual and quantitative information(6,42-44). A key finding from our review is the significant variability in methodology among studies assessing CD10. The lack of standardization in factors such as antibody clones, dilution ratios, scoring systems, and positivity thresholds greatly limits the comparability of results. These inconsistencies underscore the urgent need for consensus guidelines on CD10 assessment in gynecologic pathology. Until such standards are adopted, using CD10 as a routine diagnostic or prognostic marker will remain limited.

Limitations of the current research include inconsistent IHC techniques, which hinder reliable comparisons and conclusions regarding the prognostic value of CD10. The relationship between CD10 and tumor dedifferentiation requires further investigation, as does its potential involvement in EMT and the immune microenvironment. Additionally, small sample sizes and retrospective study designs, along with varying dilution protocols and staining thresholds, have made meta-analyses unfeasible. Future research should aim to standardize IHC protocols for CD10, clarify its role in tumor progression, evaluate its significance in larger patient cohorts, and explore its integration into multiplex biomarker panels to improve diagnostic accuracy for challenging tumor subtypes.

## Conclusion

In conclusion, CD10 is an important stromal marker with potential diagnostic value in endometrial carcinoma, particularly in endometrioid subtypes. It shows distinct spatial staining patterns that could help distinguish invasive carcinoma from benign mimics and assist in identifying mesonephric-like differentiation when used with broader immunohistochemical panels. However, differences in methodological approaches and inconsistent links to clinical outcomes limit its current usefulness. The value of CD10 may be increased when interpreted alongside other markers, such as IFITM1, p16, ER/PR, and mesonephric-associated proteins. Future research should aim to standardize immunohistochemistry techniques and investigate CD10’s role in tumor biology to fully realize its diagnostic and prognostic potential.

## Abbreviations

CD: Cluster of Differentiation

CALLA: Common Acute Lymphoblastic Leukemia Antigen

EC: Endometrial Carcinoma

IHC: Immunohistochemistry

MLA: Mesonephric-like Adenocarcinoma

NPV: Negative Predictive Value

EEC: Endometrial Endometrioid Carcinoma

FIGO: International Federation of Gynecology and Obstetrics

NR: Not Reported

APA: Atypical Polypoid Adenomyoma

CS: Carcinosarcoma

CCC: Clear Cell Carcinoma

DFS: Disease-Free Survival

TTF-1: Thyroid Transcription Factor-1

GATA3: GATA Binding Protein 3

IFITM1: Interferon-Induced Transmembrane Protein 1

PR: Progesterone Receptor

ER: Estrogen Receptor

BCL-2: B-cell lymphoma 2

HER2: Human Epidermal Growth Factor Receptor 2

PRISMA: Preferred Reporting Items for Systematic Reviews and Meta-Analyses

## Data Availability

The data that support the findings of this study are available from the corresponding author upon request.

## References

[1] Crosbie EJ, Kitson SJ, McAlpine JN, Mukhopadhyay A, Powell ME, Singh N (2022). Endometrial cancer. Lancet.

[2] McCluggage WG, Bosse T, Gilks CB, Howitt BE, McAlpine JN, Nucci MR (2024). FIGO 2023 endometrial cancer staging: too much, too soon?. FIGO 2023 endometrial cancer staging: too much, too soon? Int J Gynecol Cancer.

[3] Hosseinirad M, Mirzaeian E, Charejoo A (2025). Mesonephric-like adenocarcinoma of the ovary: A rare case report and review of the literature. Curr Probl Cancer Case Rep.

[4] Chen H, Strickland AL, Castrillon DH (2022). Histopathologic diagnosis of endometrial precancers: Updates and future directions. Semin Diagn Pathol.

[5] Wang S, Xiao Y, An X, Luo L, Gong K, Yu D (2024). A comprehensive review of the literature on CD10: its function, clinical application, and prospects. Front Pharmacol.

[6] Karamvand M, Mehrabi MM, Jalali Nadoushan M (2026). Assessing the correlation between CD15 expression and gleason score and grade groups in prostate adenocarcinoma: A cross-sectional study. Cancer Treat Res Commun.

[7] Dall'Era MA, True LD, Siegel AF, Porter MP, Sherertz TM, Liu AY (2007). Differential expression of CD10 in prostate cancer and its clinical implication. BMC Urol.

[8] Abdou AG (2007). CD10 expression in tumour and stromal cells of bladder carcinoma: an association with bilharziasis. APMIS.

[9] Ahmed ARH, Muhammad EMS (2014). E-cadherin and CD10 expression in atypical hyperplastic and malignant endometrial lesions. J Egypt Natl Canc Inst.

[10] Louhichi T, Saad H, Dhiab MB, Ziadi S, Trimeche M (2018). Stromal CD10 expression in breast cancer correlates with tumor invasion and cancer stem cell phenotype. BMC Cancer.

[11] Sumathi VP, McCluggage WG (2002). CD10 is useful in demonstrating endometrial stroma at ectopic sites and in confirming a diagnosis of endometriosis. J Clin Pathol.

[12] Groisman GM, Meir A (2003). CD10 is helpful in detecting occult or inconspicuous endometrial stromal cells in cases of presumptive endometriosis. Arch Pathol Lab Med.

[13] McCluggage WG, Sumathi VP, Maxwell P (2001). CD10 is a sensitive and diagnostically useful immunohistochemical marker of normal endometrial stroma and of endometrial stromal neoplasms. Histopathology.

[14] Barroeta JE, Pasha TL, Acs G, Zhang PJ (2007). Immunoprofile of endocervical and endometrial stromal cells and its potential application in localization of tumor involvement. Int J Gynecol Pathol.

[15] Bergman-Larsson J, Gustafsson S, Mear L, Huvila J, Tolf A, Olovsson M (2022). Combined expression of HOXA11 and CD10 identifies endometriosis versus normal tissue and tumors. Ann Diagn Pathol.

[16] Ohishi Y, Kaku T, Kobayashi H, Aishima S, Umekita Y, Wake N (2008). CD10 Immunostaining distinguishes atypical polypoid adenomyofibroma (atypical polypoid adenomyoma) from endometrial carcinoma invading the myometrium. Hum Pathol.

[17] Pors J, Cheng A, Leo JM, Kinloch MA, Gilks B, Hoang L (2018). A Comparison of GATA3, TTF1, CD10, and Calretinin in Identifying Mesonephric and Mesonephric-like Carcinomas of the Gynecologic Tract. Am J Surg Pathol.

[18] Page MJ, McKenzie JE, Bossuyt PM, Boutron I, Hoffmann TC, Mulrow CD (2021). The PRISMA 2020 statement: an updated guideline for reporting systematic reviews. BMJ.

[19] Peterson J, Welch V, Losos M, Tugwell PJ (2011). The Newcastle-Ottawa scale (NOS) for assessing the quality of nonrandomised studies in meta-analyses. The Newcastle-Ottawa scale (NOS) for assessing the quality of nonrandomised studies in meta-analyses.

[20] Busca A, Djordjevic B, Giassi A, Parra-Herran C (2016). IFITM1 is superior to CD10 as a marker of endometrial stroma in the evaluation of myometrial invasion by endometrioid adenocarcinoma. Am J Clin Pathol.

[21] Kihara A, Amano Y, Yoshimoto T, Matsubara D, Fukushima N, Fujiwara H (2019). Stromal p16 Expression Helps Distinguish Atypical Polypoid Adenomyoma from Myoinvasive Endometrioid Carcinoma of the Uterus. Am J Surg Pathol.

[22] Kim H, Na K, Bae GE, Kim HS (2021). Mesonephric-like adenocarcinoma of the uterine corpus: Comprehensive immunohistochemical analyses using markers for mesonephric, endometrioid and serous tumors. Diagnostics (Basel).

[23] Lee Y, Choi S, Kim HS (2024). Comprehensive Immunohistochemical Analysis of Mesonephric Marker Expression in Low-grade Endometrial Endometrioid Carcinoma. Int J Gynecol Pathol.

[24] Ma T, Chai M, Shou H, Ru G, Zhao M (2022). Mesonephric-Like Adenocarcinoma of Uterine Corpus: A Clinicopathological and Targeted Genomic Profiling Study in a Single Institution. Front Oncol.

[25] McCluggage WG, Oliva E, Herrington CS, McBride H, Young RH (2003). CD10 and calretinin staining of endocervical glandular lesions, endocervical stroma and endometrioid adenocarcinomas of the uterine corpus: CD10 positivity is characteristic of, but not specific for, mesonephric lesions and is not specific for endometrial stroma. Histopathology.

[26] Nascimento AF, Hirsch MS, Cviko A, Quade BJ, Nucci MR (2003). The role of CD10 staining in distinguishing invasive endometrial adenocarcinoma from adenocarcinoma involving adenomyosis. Mod Pathol.

[27] Norimatsu Y, Nishikawa T, Suzuki H, Hosokawa S, Yano H, Maeda Y (2022). The expression pattern of CD10 and CD31 identifies fine fibrovascular stroma of grade 1-endometrial endometrioid carcinomas in cytology. Cytopathology.

[28] Srodon M, Klein WM, Kurman RJ (2003). CD10 immunostaining does not distinguish endometrial carcinoma invading myometrium from carcinoma involving adenomyosis. Am J Surg Pathol.

[29] Suzuki T, Kikkawa F, Ino K, Nagasaka T, Tamakoshi K, Mizutani S (2001). Imbalance between neutral endopeptidase 24.11 and endothelin-1 expression in human endometrial carcinoma. Oncology.

[30] Youssef MY, Mohamed MA (2019). Could E-Cadherin and CD10 Expression be Used to Differentiate between Atypical Endometrial Hyperplasia and Endometrial Carcinoma?. Could E-Cadherin and CD10 Expression be Used to Differentiate between Atypical Endometrial Hyperplasia and Endometrial Carcinoma? Int J Gynecol Pathol.

[31] Murray SK, Clement PB, Young RH (2005). Endometrioid carcinomas of the uterine corpus with sex cord-like formations, hyalinization, and other unusual morphologic features: A report of 31 cases of a neoplasm that may be confused with carcinosarcoma and other uterine neoplasms. Am J Surg Pathol.

[32] Chekmareva M, Ellenson LH, Pirog EC (2008). Immunohistochemical differences between mucinous and microglandular adenocarcinomas of the endometrium and benign endocervical epithelium. Int J Gynecol Pathol.

[33] Bilalovic N, Sandstad B, Golouh R, Nesland JM, Selak I, Torlakovic EE (2004). CD10 protein expression in tumor and stromal cells of malignant melanoma is associated with tumor progression. Mod Pathol.

[34] Kumar R, Chadha S, Ruhela S, Agarwal S, Singhal S (2024). Expression of CD10 in invasive breast carcinoma and its correlation with other clinicopathological parameters. Int J Acad Med Pharm.

[35] Suzuki T, Ino K, Kikkawa F, Uehara C, Kajiyama H, Shibata K (2002). Neutral endopeptidase/CD10 expression during phorbol ester-induced differentiation of choriocarcinoma cells through the protein kinase C-and extracellular signal-regulated kinase-dependent signalling pathway. Placenta.

[36] Pradhan S, Bajad C, Mishra DP, Dash A, Chowdhary S, Behera SK (2017). Stromal expression of CD10 in invasive breast carcinoma and its correlation with known prognostic markers. J Evid Based Med Healthc.

[37] Zhao W, Cui M, Zhang R, Shen X, Xiong X, Ji X (2021). IFITM1, CD10, SMA, and h-caldesmon as a helpful combination in differential diagnosis between endometrial stromal tumor and cellular leiomyoma. BMC Cancer.

[38] Toki T, Shimizu M, Takagi Y, Ashida T, Konishi I (2002). CD10 is a Marker for Normal and Neoplastic Endometrial Stromal Cells. Int J Gynecol Pathol.

[39] Puri V, Jain M, Thomas S (2011). Stromal Expression of CD10 in Invasive Breast Carcinoma and Its Correlation with ER, PR, HER2-neu, and Ki67. Int J Breast Cancer.

[40] Żurawski J, Talarska P, de Mezer M, Kaszkowiak K, Chalcarz M, Iwanik K (2022). Evaluation of CD10 expression as a diagnostic marker for colorectal cancer. Gastroenterol Hepatol Bed Bench.

[41] Bahadir B, Behzatoglu K, Bektas S, Bozkurt ER, Ozdamar SO (2009). CD10 expression in urothelial carcinoma of the bladder. Diagn Pathol.

[42] Cregger M, Berger AJ, Rimm DL (2006). Immunohistochemistry and quantitative analysis of protein expression. Arch Pathol Lab Med.

[43] Mebratie DY, Dagnaw GG (2024). Review of immunohistochemistry techniques: Applications, current status, and future perspectives. Semin Diagn Pathol.

[44] Ebrahimzadeh Pasha A, Ehsani A, Ashtari S, Najafi MS, Baik B, Mehrabi MM (2026). An In-Depth Evaluation of CD15 Expression in Thyroid Carcinoma: A Comprehensive Systematic Review. Cancer Rep.

